# Detection of Rare G3P[19] Group A Rotavirus in Human Patient, Italy

**DOI:** 10.3201/2011.131699

**Published:** 2014-11

**Authors:** Giovanni Ianiro, Roberto Delogu, Rosalia Graffeo, Maurizio Sanguinetti, Lucia Fiore, Franco M. Ruggeri

**Affiliations:** Istituto Superiore di Sanità, Rome, Italy (G. Ianiro, R. Delogu, L. Fiore, F.M. Ruggeri);; University Hospital “Agostino Gemelli,” Rome (R. Graffeo, M. Sanguinetti)

**Keywords:** group A rotavirus, diarrhea, gene, evolution, sequence, G3P[19], human, adult, Italy, viruses, rotavirus

## Abstract

Infection with a rare G3P[19] rotavirus A strain was identified in an immunosuppressed patient in Italy. The strain showed a P[19] viral protein 4 gene and a complete AU-1–like genomic constellation. Phylogenetic analyses showed high nucleotide identity between this strain and G3P[19] rotavirus A strains from Asia, indicating possible reassortment events.

Group A rotavirus (RVA) is the leading cause of acute gastroenteritis in children <5 years of age worldwide, causing ≈450,000 deaths annually. The RVA genome is composed of 11 double-stranded RNA segments, encoding 6 structural viral (VP) and 5 nonstructural (NS) proteins (*1*). The outer capsid proteins, VP7 and VP4, elicit neutralizing antibodies. The genes encoding these proteins specify at least 27 G and 37 P genotypes, which are used for RVA binary classification. 

Most RVA human infections worldwide are related to 5 major genotypes: G1P[8], G2P[4], G3P[8], G4P[8], and G9P[8] (*2*). Genome segment reassortment between human strains or human and animal strains during co-infections can generate viruses with novel genotype combinations, possibly influencing the virus phenotype (*2*). Some human and animal RVA strains possess unusual genotype combinations (*3*,*4*), and some strains might partially escape vaccine-induced immune protection (*5*).

Since 2007, the RVA surveillance network RotaNet-Italy has confirmed circulation of common RVA genotypes among children in Italy, despite sporadic uncommon, exotic, or zoonotic genotypes (*6*,*7*). We describe infection with a rare G3P[19] RVA strain in an immunosuppressed adult patient in Italy who had severe diarrhea. 

## The Study

In 2012, a 35-year-old woman who was hospitalized in the Hematology Unit of Rome University Hospital “Agostino Gemelli” in Rome, Italy; she experienced acute gastroenteritis after a bone marrow allotransplant. Stool samples were collected and tested for classic bacterial, viral, and parasitic enteropathogens. The study was performed in compliance with informed consent guidelines in Italy. 

Viral RNA was extracted by using the Viral RNeasy MiniKit (QIAGEN/Westburg, Milan, Italy) and stored at −80°C until use. Rotavirus G- and P-genotyping were performed by reverse transcription nested PCR by using VP7 or VP4 primer mixtures described previously (*8*,*9*). Nucleotide sequencing was performed by Macrogen, Inc. (Seoul, South Korea) by using the PCR primers. After analysis in Chromas Pro 2.23 (http://www.technelysium.com.au), consensus sequences were obtained by using SeqMan II (http://www.dnastar.com/t-seqmanpro.aspx). Multiple sequence alignments were carried out, and phylogenetic trees were created by using MEGA5 software (http://www.megasoftware.net) (*10*), using the maximum-likelihood method and Kimura 2- (NS 4–5) or Tamura 3- (all other genes) parameter tests. Strain sequences from this study were deposited in GenBank (accession nos. KF729023–729032).

The patient had Down syndrome, acute lymphatic leukemia, and blood type A Rh+ CCDeekk phenotype; a transcranial Doppler scane did not show any abnormalities . She had received a stem cell allotransplant, followed by immunosuppressive treatment. Acute gastroenteritis began 2 days after immunosuppression, on day 10 after admission to the Hematology Unit. Diarrhea was nonbloody and watery, not accompanied by vomiting and fever, and lasted 3 days, during which rehydration therapy was administered. The patient was released from the hospital in stable condition; she died of systemic complications 3 months later.

Stool samples were collected at diarrhea onset and tested for bacterial and viral enteric pathogens. Results were negative for *Salmonella, Shigella, Campylobacter, Yersinia*, *Escherichia coli*, staphylococci, *Giardia*, norovirus, and adenovirus. Only rotavirus and *Klebsiella pneumoniae* were detected; because the patient did not exhibit chronic/bloody diarrhea or other systemic pathologies typically related to *K. pneumoniae* infection, this pathogen was not investigated further. 

**Figure 1:**
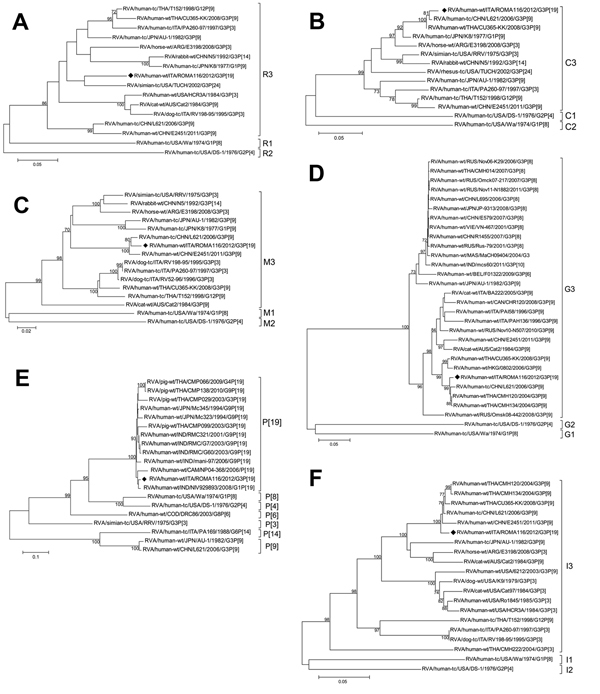
Phylogenetic trees of rotavirus A (RVA) isolates based on the open reading frames of genes coding for the viral protein (VP) regions. A) VP1 (nt 73–390); B) VP2 (nt 1–425); C) VP3 (nt 44–880); D) VP7 (nt 48–1029); E) VP4 (nt 36–817); F) VP6 (nt 44–1326). The G3P[19] strain ROMA116 identified from the patient in Italy described in this article is highlighted with a black diamond. Trees were built with the maximum likelihood method and bootstrapped with 1,000 repetitions; bootstrap values <70 are not shown. Scale bars indicate nucleotide substitutions per site. Letters and numbers on the right indicate the specific gene genotype (http://rotac.regatools.be/classificationinfo.html).

**Figure 2:**
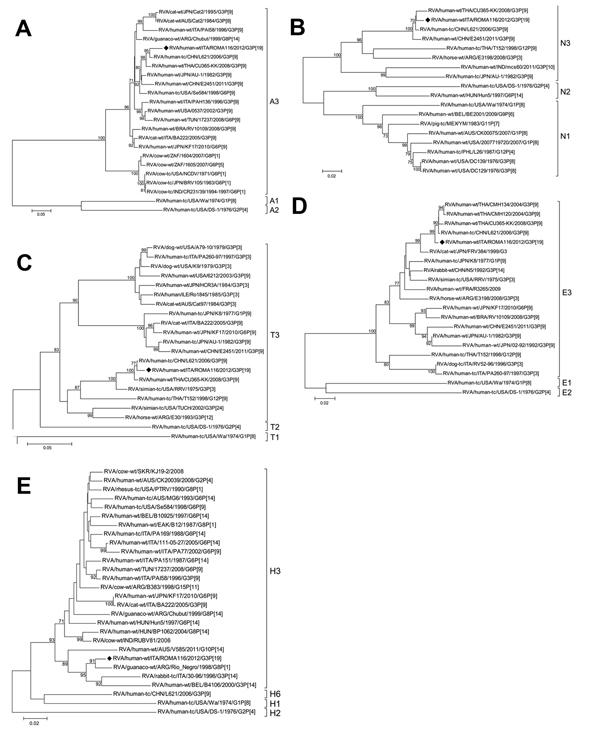
Phylogenetic trees of rotavirus A (RVA) isolates based on the open reading frames of genes coding for the nonstructural protein (NS) regions. A) NS1 (nt 67–1087), B) NS2 (nt 47–1012), C) NS3 (nt 31–1049), D) NS4 (nt 49–660), and E) NS5 (nt 63–627). The G3P[19] strain ROMA116 identified from the patient in Italy described in this article is highlighted with a black diamond. Trees were built with the maximum likelihood method and bootstrapped with 1,000 repetitions; bootstrap values <70 are not shown. Scale bars indicate nucleotide substitutions per site. Letters and numbers on the right indicate the specific gene genotype (http://rotac.regatools.be/classificationinfo.html).

The rotavirus strain, RVA/human-wt/ITA/ROMA116/2012/G3P[19] (ROMA116), was characterized by analyzing its 11 genomic RNA segment sequences in RotaC Tool (http://rotac.regatools.be/). The strain showed the genotype constellation of G3-P[19]-I3-R3-C3-M3-A3-N3-T3-E3-H3. Phylogenetic analyses confirmed a full AU-1–like genomic constellation, associated with the P[19] VP4 gene (Figures 1, 2). The strain clustered strictly with RVA/human-tc/CHN/L621/2006/G3P[9] from China (*11*), sharing 98%–99% nucleotide identities for most genes except VP1 (identity 91%), and the VP4 and NS5 genes, which belonged to different genotypes (Figures 1, 2). ROMA116 also showed high nucleotide identities (98%–99%) in VP2, VP6–7, and NS1–4 genes with strain RVA/human-wt/THA/CU365-KK/2008/G3P[9] from Thailand (*12*).

The VP7 tree (Figure 1, panel D) revealed strict clustering of ROMA116 with G3 strains from China, Thailand, and Hong Kong, all associated with P[9] VP4. However, other G3 RVA strains from Italy reported in humans or cats grouped in the same cluster. The VP4 tree (Figure 1, panel E) shows the correlation of the ROMA116 P[19] sequence with P[19] sequences detected in human and swine strains from 1994–2010, suggesting possible human-pig reassortment at the origin of ROMA116 VP4. Further evidence of reassortment resulted from both VP1 and NS5 tree analyses. In VP1, ROMA116 showed the highest nucleotide identity (95%) with simian strain TUCH (Figure 1, panel A); in NS5, the uncommon H3 genotype of ROMA116 clustered with strains detected in or derived from animals (Figure 2, panel E).

The phylogenetic trees show the divergence of ROMA116 from the constellation 3 putative ancestor AU-1 (*13*), characterized during the 1980s. ROMA116 shared relatively high nucleotide sequence conservation of only the NS1 gene with AU-1, but all other genes analyzed clustered more closely with RVA strains detected in Asia. This mixed genomic pattern probably was generated by previous reassortment events between strains circulating in that area. Analysis of the VP1, VP4, and NS5 gene trees together indicates that ROMA116 may have evolved through multiple reassortment events involving RVA strains of different animal origins.

## Conclusions

The G3P[19] RVA strain we identified represents a single sporadic detection among >7,000 human RVA strains investigated in Italy during a 7-year period, which suggests either a recent introduction or a low ability of this strain to spread among humans. However, the phylogenetic analysis shows that the overall genome of ROMA116 is more similar to those reported for human strains than for animal strains, suggesting that the strain has a lower fitness for replicating in animal hosts than in humans. A study in Thailand (*14*) reported an outbreak of diarrhea in piglets caused by G3P[19] RVA, but no information was available for the other genes of that strain.

The possible importation of an apparently exotic rotavirus strain such as ROMA116 into Italy is not surprising; the country’s geographic position favors massive migratory flows of persons from developing countries. Although rare, similar events have been suggested previously (*7*). The source of this infection was not identified; no additional case was reported among hospital ward patients and personnel or in the patient’s family. The patient’s parents had been cleared to assist their daughter daily after the transplant, but strict control measures for opportunistic infectious agents were otherwise enforced. The patient’s family lived in a rural area where swine, bovine, and ovine farming activities occur in close proximity to human residential settlements, which may favor the circulation and zoonotic transmission of viruses from domestic animals to a higher extent than is possible inside urban settings such as Rome. The G3P[19] RVA strain may have been transmitted by an asymptomatic but infected relative, or the patient may have been harboring the strain in the gut before hospital admission, with active viral replication and disease occurring after immunosuppressive treatment.

Because no other enteropathogens were detected among the large panel of bacteria, viruses, and parasites investigated, it is likely that rotavirus was directly involved in causing illness in the patient, whose clinical symptoms were compatible with acute watery rotavirus diarrhea. It is possible that this RVA genotype may not cause disease in immunocompetent persons and that the compromised immune status of this patient played a critical role. Even if G3P[19] RVA, as with other uncommon viral strains, does not present a direct risk for public health in Italy, it could nonetheless be a donor of atypical RVA genes that might reassort into novel epidemic strains that could escape existing herd immunity in humans. In this view, RVA surveillance of both farmed and pet animals could be of valuable support to human surveillance of severe cases in hospitals (*15*), particularly in the postvaccine globalized world.
